# Advances in ion mobility spectrometry–mass spectrometry reveal key insights into amyloid assembly^☆^

**DOI:** 10.1016/j.bbapap.2012.10.002

**Published:** 2013-06

**Authors:** L.A. Woods, S.E. Radford, A.E. Ashcroft

**Affiliations:** Astbury Centre for Structural Molecular Biology & School of Molecular and Cellular Biology, University of Leeds, LS2 9JT, UK

**Keywords:** CCS, collision cross-section, ESI–MS, electrospray ionization–mass spectrometry, HDX, hydrogen–deuterium exchange, IMS–MS, ion mobility spectrometry–mass spectrometry, Amyloid, Ion mobility spectrometry, Mass spectrometry, Oligomer, Ligand binding

## Abstract

Interfacing ion mobility spectrometry to mass spectrometry (IMS–MS) has enabled mass spectrometric analyses to extend into an extra dimension, providing unrivalled separation and structural characterization of lowly populated species in heterogeneous mixtures. One biological system that has benefitted significantly from such advances is that of amyloid formation. Using IMS–MS, progress has been made into identifying transiently populated monomeric and oligomeric species for a number of different amyloid systems and has led to an enhanced understanding of the mechanism by which small molecules modulate amyloid formation. This review highlights recent advances in this field, which have been accelerated by the commercial availability of IMS–MS instruments. This article is part of a Special Issue entitled: Mass spectrometry in structural biology.

## Graphical abstract

**Figure:**
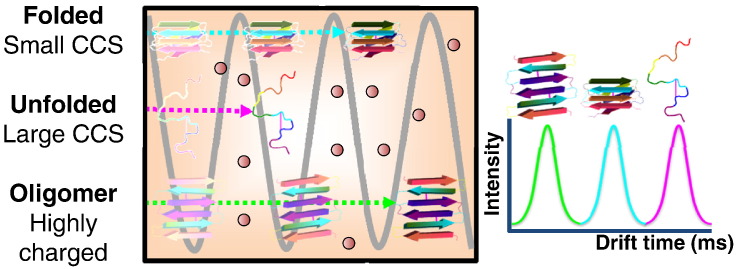

**Highlights:**

► IMS–MS provides insights into the mechanism of amyloid formation. ► IMS–MS enables individual conformers of unfolded proteins to be characterized. ► IMS–MS separates and characterizes individual oligomers in a heterogeneous ensemble. ► Ligand binding to specific protein conformers can be detected using IMS–MS.

## 1 Introduction

Mass spectrometry (MS) has become widely accepted as a tool to analyse biological systems over the past twenty years, subsequent to the pioneering development of electrospray ionization (ESI) [1,2]. The advent of ESI, and other soft ionization techniques such as matrix assisted laser desorption ionization (MALDI) [3], initiated a new field in which a wealth of information about protein structure and macromolecular assemblies can be deduced using MS, including revealing insights about protein/ligand binding and dynamic structure. Whereas the first ESI–MS experiments of biological macromolecules provided accurate molecular mass measurements [2], the technique evolved rapidly such that structural information about proteins and their biomolecular complexes could be obtained, in addition to information about protein stability, dynamics and post-translational modifications [4–12]. The use of ESI–MS in structural biology has been accelerated most recently through the coupling of ion mobility spectrometry (IMS) to MS [13–16]. This approach has enabled the detailed analysis of biological systems, particularly in cases where alternative techniques such as crystallography or nuclear magnetic resonance are unable to be used due to the analyte's poor solubility, large mass, and/or the inability to crystallize, or in cases where the species of interest are present within a heterogeneous mixture [17]. In this review, we describe the principles of mass spectrometry coupled with ion mobility spectrometry (IMS–MS) and discuss how this approach has enhanced our understanding of protein oligomerization during self-assembly into amyloid.

## 2 Background to ESI–IMS–MS development and application to biological systems

Ion mobility spectrometry (IMS) is able to separate complex mixtures of ions based on their shape and/or charge [18,19], yielding structural information complementary to molecular mass measurements. The technique of IMS relies on separating gaseous ions according to their mobility through a drift-tube filled with a buffer gas [18]. Ions are accelerated through the drift tube by an electric field, wherein ions of differing shapes have different mobilities depending on an ion's characteristic collision cross-section (CCS). Larger, extended ions will experience more collisions with the buffer gas and, as a result, will take longer to traverse the drift tube in comparison with smaller, more compact ions of the same molecular mass which will undergo fewer collisions with the buffer gas and hence will have a greater mobility and a shorter drift time. One caveat to ion mobility analysis is that the CCS data collected from ion mobility analyses give a rotationally averaged value, as the gaseous ions are able to collide with the buffer gas in a number of different orientations. Despite this, when used in conjunction with mass spectrometry, it has been shown that structural features of proteins and protein complexes can be elucidated [20,21].

**Fig. 1:**
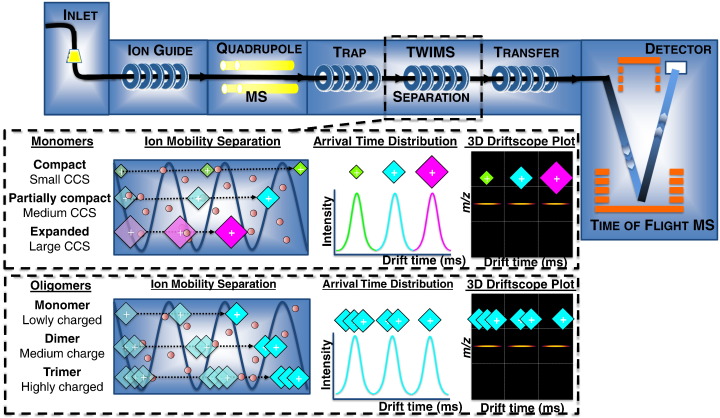
Schematic showing commercially available travelling-wave ion mobility spectrometry (TWIMS) integrated with an orthogonal acceleration quadrupole-time-of-flight mass spectrometer. Ions are separated in the TWIMS device based on their mobility through the drift cell; ions with a large collision cross-section (CCS) experience more collisions with the buffer gas molecules present in the drift cell and have a longer drift time than more compact ions of the same mass and same charge. Ions of the same *m*/*z* but different mass and different charge, for example a mixture of protein oligomers including a monomer with one charge, a dimer with two charges etc., are separated based on both their CCS and their number of charges. Typically, for a given *m*/*z*, the more highly charged ions have a shorter drift time, as they are propelled through the drift cell faster.

The use of conventional ion mobility drift tubes coupled to MS has produced excellent results but has been limited to home-built instrumentation [16,22–24]. Over the past decade, much effort has been directed at producing commercially viable instrumentation. This has led to the development of ion mobility separation based on either a differential IMS setup [25,26] or a travelling-wave IMS system (Fig. 1) [15]. In particular, travelling-wave IMS–MS has been found to be promising for protein analysis, showing good sensitivity and separative power, and it can be used to measure the mass and to estimate CCS value for each individual component within a sample. Although it is possible to acquire CCS values directly from drift-times using conventional IMS [27], calibration of the travelling-wave IMS device with ions of known CCS is required [28–30]. Once the travelling-wave IMS device has been calibrated, CCSs can be estimated for previously uncharacterised species. In the case of a protein, a CCS can be derived for each of its charge state ions. A folded, native-like protein conformer may only give rise to two or three charge state ions, all with similar CCSs; however, an unfolded protein conformer could generate a wide charge state distribution, with the higher charge state ions having much larger CCSs than the lower charge state ions, a phenomenon due in part to Coulombic repulsions between the charges of the highly charged ions. Therefore, for each protein conformer, the CCS of the lowest charge state ions is usually the smallest and therefore the one used to define that particular conformer. Using this approach, IMS–MS has revealed valuable information on a range of biological systems [31–34]. One area that has benefited greatly from such analyses is that in which transient intermediate species are lowly populated and short lived, such as in amyloid fibril formation, as discussed below.

## 3 Amyloidosis: a challenging biomolecular assembly system ideal for analysis by ESI–IMS–MS

**Fig. 2:**
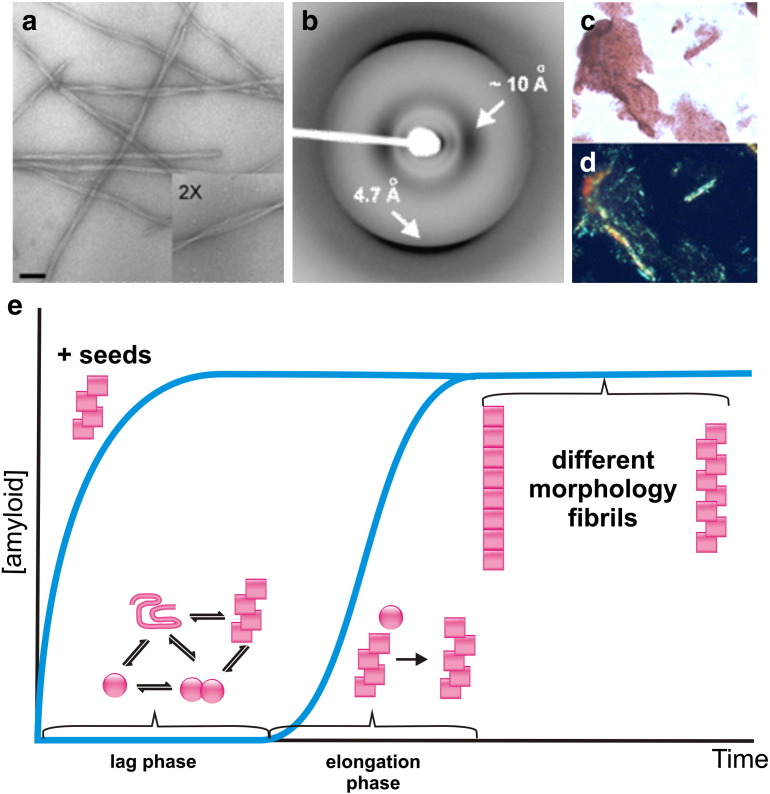
Characterisation of in vitro β_2_-microglobulin (β_2_m) amyloid fibril formation. (a) Electron microscopy image of long, straight β_2_m fibrils (scale bar = 100 nm); (b) X-ray fibre diffraction of long, straight β_2_m fibrils showing the hallmark amyloid pattern; (c) Congo red staining and (d) Congo red birefringence of β_2_m fibrils. (e) Schematic representation of fibril formation starting from monomer (pink circles) and proceeding via dimers and higher-order oligomers (pink squares) along seeded and unseeded assembly pathways to produce fibrils of different morphologies.

More than 25 different proteins or peptides, whose aggregation into fibrils is associated with amyloidosis in vivo, have been identified to date [35]. In different amyloid disorders, fibrils accumulate in specific regions of the body causing the variety of symptoms associated with individual diseases [36,37]. Due to the prevalence of these diseases, gaining insights into how amyloid deposits form and how their production can be halted is crucial. To achieve this, in vitro techniques have been employed to provide information. The advantage of such experiments is that amyloid formation can be induced to occur on relatively rapid timescales, rather than the decades or more generally required for amyloid to become symptomatic in vivo. Accordingly, decreasing the pH, incubating with protein denaturants such as sodium dodecyl sulphate, exposing the protein or peptide to metal ions, introducing destabilising mutations into the protein sequence, and/or agitating the solution vigorously have all been used to initiate rapid fibril formation in vitro [37–39]. The formation of amyloid-like fibrils under these conditions can be confirmed using imaging methods such as electron microscopy (Fig. 2a) or atomic force microscopy [35], complemented by X-ray fibre diffraction to confirm the presence of the well-characterized cross-β structure of amyloid (Fig. 2b) [37]. Additionally, small organic molecules known to bind to amyloid fibrils are commonly used to detect their presence: Congo red is particularly useful in this role as upon excitation with plane-polarised light the dye exhibits apple-green birefringence when bound to the regular array of β-strands within the cross-β structure of amyloid [40] (Fig. 2c, d). Thus, Congo red birefringence is an excellent diagnostic tool for indicating the presence of amyloid fibrils formed in vitro or in vivo. In addition, the dye thioflavin T is commonly used to monitor fibril formation both in vitro and in vivo, as a change in fluorescence of this molecule is experienced upon binding to fibrils [41]. In in vitro experiments, thioflavin T fluorescence can be monitored in real-time during protein aggregation to determine the kinetics of fibril formation. Typically, ordered amyloid assembly proceeds via a nucleated assembly mechanism [42,43], consisting of a lag-phase in which a critical nucleus forms, followed by a growth phase and stationary phase (Fig. 2e). Identification of species in each of these phases is crucial for understanding the mechanism(s) of fibril formation and for identifying potential targets for ligand binding. The latter is especially important as such studies could pave the way for the development of therapeutics to block fibril formation and/or control oligomer production with its associated toxicity.

Protein aggregation of an initially soluble protein monomer is thought to be initiated by a misfolding or unfolding event, resulting in one or more species capable of undergoing oligomerization. Next, oligomers form and may either assemble directly into amyloid fibrils or, alternatively, undergo a structural rearrangement preceding fibril formation [44,45]. Identifying oligomeric species both on- and off-pathway to fibril formation is challenging, yet crucial to understanding the molecular mechanisms of fibrillogenesis. While using small molecules to target on-pathway oligomeric species could lead to the inhibition of fibril formation, off-pathway oligomers are equally interesting as in some cases they have been shown to exhibit toxicity against numerous cell-lines [46,47]. It is for these reasons that research into fibril formation is divided between those who consider amyloid fibrils to be the primary cause of the symptoms associated with disease and those who consider the oligomers present during fibril formation, or alternatively formed by dissociation of fibrils, to be the causative agents of the disease [48–51]. Both such scenarios could be involved, at least in some disorders. Therefore, it is essential to identify all of the monomeric and oligomeric species present within these heterogeneous mixtures, and to characterize their structures, dynamic properties and ligand binding capabilities of each as much as possible. It is here that mass spectrometry can excel.

## 4 ESI–IMS–MS as a separative technique for characterizing amyloidogenic conformers

ESI–IMS–MS is particularly useful in the study of amyloid fibril formation, being capable of separating co-populated multiple conformations of the same protein monomer with the same *m*/*z* ratio but that differ in physical size or shape. This is a key issue in amyloid fibril formation as protein unfolding or mis-folding frequently precedes fibril formation and hence the conformational properties of the protein require characterization. Numerous studies have shown that the electrospray process can conserve protein's solution-phase characteristics in the gas-phase and, using ESI–MS alone, it is possible to estimate the population of different conformational states by interpretation of the protein's charge state distribution [4,52–55]. Unfolded conformations expose more ionizable sites and thus give rise to higher charge states during ESI than more folded, and therefore more compact, conformations of the same protein. Using this approach, Gaussian distributions can be fitted to the charge state envelopes observed in a protein's *m*/*z* spectrum to estimate the number of conformers (or conformeric families) present [4,7,52,53,55]. For example, in the case of α-synuclein, an intrinsically unstructured, highly amyloidogenic, 14.5 kDa protein associated with Parkinson's disease, multiple distinct conformations of the protein monomer have been identified using ESI–MS [53,54]. Recently, an amyloidogenic auto-proteolysis product of α-synuclein, consisting of residues 70–140, has been identified using ESI–IMS–MS [56]. This experiment involved separating the degraded α-synuclein sample from intact α-synuclein in the IMS cell, allowing the two species to be characterized individually.

Fitting charge state distributions to ESI–MS *m*/*z* spectra provides information about the number of distinct conformations present and their relative populations, but often the charge state ions of the various conformers overlap and also this approach does not allow further, individual interrogation of these species. By contrast, using ESI–IMS–MS, monomeric conformers with different CCSs can be fully separated and further information gained on each species following interpretation of their CCS. Early experiments utilising travelling-wave IMS–MS to identify amyloid precursor states were performed on beta-2 microglobulin (β_2_m), a 99-residue, seven β-stranded protein [57]. β_2_m fibril formation is of particular interest, as incubating wild-type human β_2_m alone at near-physiological pH in vitro is insufficient to cause aggregation [58,59]. However, in vivo β_2_m is the main component of fibrils deposited in patients undergoing dialysis due to renal failure. These fibrils are associated with the disease dialysis-related amyloidosis (DRA) [60,61]. It is only by modifying the protein [38,59,62–65], lowering the pH [66,67] or incubating the protein with certain additives [68–72] that fibril formation is able to proceed. In the experiments performed by Smith et al., low pH was used to initiate fibril formation [57]. Under these conditions, three different conformeric families of the protein monomer were separated using ESI–IMS–MS: compact, partially compact and expanded conformations [57], consistent with previous results obtained by fitting the charge state distributions [52]. Despite the denaturing conditions used, the compact conformer has a CCS value that correlates well with that of the native structure of β_2_m [28], as characterized by both NMR [38,73] and X-ray diffraction [74]. These results show that residual interactions are maintained within the structure of β_2_m, even under harsh solution conditions, and it has been demonstrated that this structure is necessary for fibril formation to occur [75,76].

**Fig. 3:**
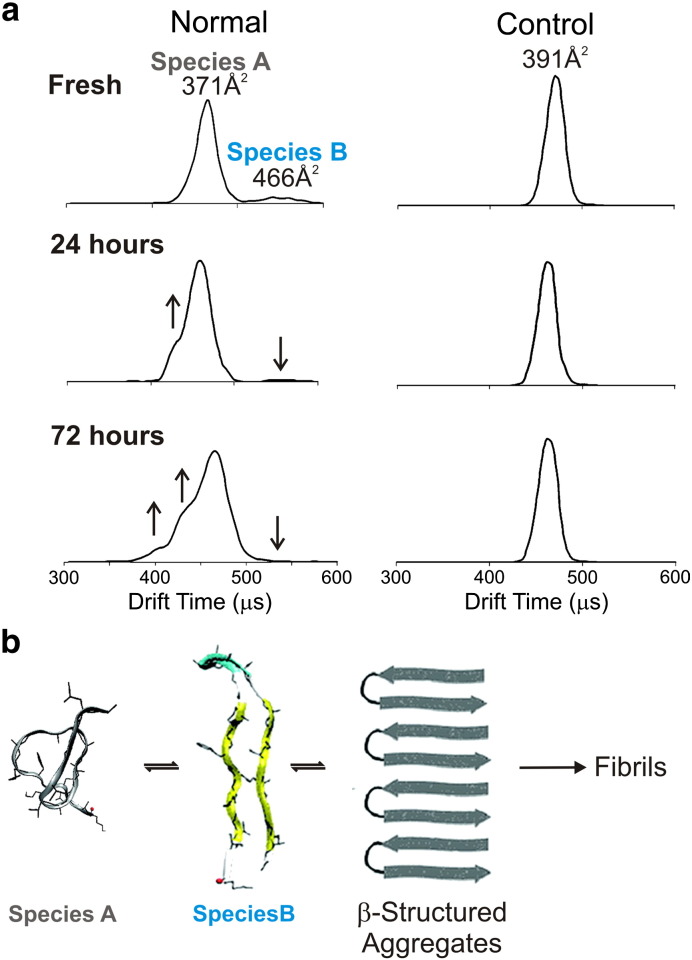
IMS–MS interrogation of the assembly pathway of the prion protein fragment 106–126 (PrP106–126) [80]. (a) Ion mobility traces for the + 2 charge state monomer ions of PrP106–126 (labeled “Normal”) showing the presence of Species A (CCS = 371 Å^2^) together with Species B, an expanded β-sheet conformation with a CCS of 466 Å^2^, at early time-points. Species B is absent in the “Control” sample, a non-amyloidogenic peptide of the same amino acid composition but with a scrambled sequence (CCS = 391 Å^2^). The population of expanded conformers in the “Normal” PrP106–126 decreases over time as species with drift times of 390 μs (CCS = 302 Å^2^) and 425 μs (CCS = 347 Å^2^) appear (see arrows). These peaks are presumed to be oligomeric as the CCS values are too small to belong to a monomeric conformer with + 2 charges. (b) Schematic showing the proposed mechanism of PrP106–126 oligomer formation involving the assembly of Species A and Species B into ordered small aggregates.

Reprinted with permission from M. Grabenauer, C. Wu, P. Soto, J.-E. Shea, M.T. Bowers, Oligomers of the Prion protein fragment 106–126 are likely assembled from β-hairpins in solution, and methionine oxidation inhibits assembly without altering the peptide's monomeric conformation, J. Am. Chem. Soc., 132 (2009) 532–539 [80].

Once the CCS of a protein conformer has been estimated it is possible to use coarse-grain modelling or molecular dynamics simulations to reveal potential structures consistent with the ESI–IMS–MS data obtained. This approach has been used in the analysis of the amyloidogenic proteins α-synuclein [77], Aβ [78], islet amyloid polypeptide (IAPP) [79], and a short peptide associated with prion aggregation [80]. In the case of IAPP fibril formation, which is associated in vivo with type II diabetes [81], the 37-residue peptide monomer undergoes a distinct change in conformation which has been identified using IMS–MS [79]. Although human IAPP is able to form fibrils spontaneously in vitro at pH 7, rat IAPP (which differs by only six residues in the core amyloidogenic region of the peptide sequence) is not amyloidogenic under the same conditions [82]. Therefore, the rat protein is ideal to use as a control [83]. Using ESI–IMS–MS, human IAPP was shown to populate a more expanded conformation than its rat counterpart [79]. Molecular dynamics simulations revealed that the expanded conformation of human IAPP is likely populated by a β-hairpin structure, whereas the more compact conformation occupied by rat IAPP can be attributed to a more helical conformation. These results are in good agreement with NMR data that identified the initiation of human IAPP fibril formation from a β-hairpin intermediate [84]. These studies testify to the use of molecular dynamics simulations restrained with CCS values determined from IMS–MS measurements to analyse the early stages of protein self-assembly into amyloid. A β-hairpin structure for the human IAPP dimer has also been suggested by combining IMS–MS with molecular dynamics simulation; CCS values were consistent with a species containing two β-hairpin IAPP monomers bound side-to-side [85]. In another example using this approach, the prion fragment 106–126 was also shown to adopt an expanded conformation, with molecular dynamics simulations again suggesting a β-hairpin structure [80]. Importantly, this conformation was absent in control samples (Fig. 3). Over time, the population of the β-hairpin-like species decreased, while ions corresponding to a second peak in the ion mobility spectrum increased. The latter species was thought to be populated by oligomers, with an increase in β-sheet structure, consistent with circular dichroism analysis.

In summary, although still in infancy compared with other structural methods, IMS–MS is now accepted as a powerful method to determine conformational properties of unfolded and partially folded species, as well as natively folded proteins. In the context of amyloid formation, this is of significance as the onset of fibril formation can be induced by rarely-populated conformers of highly dynamic proteins.

## 5 Using ESI–IMS–MS to probe the structure of oligomeric intermediates in amyloid assembly

In addition to providing insights into the structures of protein monomers, CCS values can also be assigned to larger, non-covalently bound biomolecular species [8,17,29]. A major challenge when analysing the pathway of amyloid fibril formation is identifying and characterizing transient oligomeric intermediates en route to larger aggregates and fibrils (Fig. 2e). As these early stages in amyloid formation are highly heterogeneous with multiple, rapidly inter-converting species being co-populated in solution, this presents a significant challenge. In this scenario mass spectrometry is an ideal technique: ESI–MS has been shown to be capable of preserving non-covalently bound species [5,6] and the high sensitivity of modern mass spectrometers enables detection of species present even at femtomolar concentrations within complex mixtures. Whereas ion mobility separation of protein monomers is primarily dictated by the individual shapes of the protein's conformers, the effect of charge is more predominant when separating oligomeric species arising from the same protein monomer. For example, when singly charged monomer, doubly charged dimer and triply charged trimer ions all populate the same *m*/*z*, the trimer is more likely to have a shorter drift time than the monomer, despite the trimer having a larger CCS, due to the effect of multiple charging (Fig. 1). This is an important factor to take into account when assigning oligomeric species. However, because the drift time depends on both CCS and charge, predicting the arrival time of an ion is complex and analysis of a charge state series of ions is needed to make a clear assignment for each individual species [57].

One advantage that mass spectrometry possesses over other biophysical techniques is its ability to distinguish between individual oligomeric states within highly heterogeneous systems. By contrast, techniques such as light-scattering or fluorescence reveal only information about the average properties of the species populated. Single molecule fluorescence, especially when combined with two colour coincidence detection, can separate different oligomeric species but requires them to have significant differences in their diffusion times [51,86,87], and even so, this technique has a resolution much poorer than that of IMS–MS. Early ESI–MS experiments have identified high-order oligomeric species for several different amyloid systems [88,89]. In these examples, oligomer size was determined using *m*/*z* alone. This approach relies on identifying charge states based on the observed C^13^ isotope spacing [90], or identifying oligomer size based on unique, odd numbered charge states [88,89]. However, in some cases it is impossible to establish whether a particular oligomer is present using ESI–MS alone, especially where the ions of a particular species have *m*/*z* values that all coincide with those from other oligomers. Further separation is required to confirm the repertoire of oligomers populated during fibril formation. Using ESI–IMS–MS this is possible [80,85,91–95]; ions with *m*/*z* values that have contributions from multiple different oligomeric states can be separated and investigated in terms of structure and dynamics due to differences in their mobility as they travel through the IMS drift cell.

The assemblies of Aβ_40_ and Aβ_42_ have been characterized extensively using ESI–IMS–MS [78,90,91,95,96]. The peptide Aβ is cleaved from the amyloid precursor protein (APP), an event which produces predominantly either a 40- or 42-residue peptide (Aβ_40_ and Aβ_42_) [49]. Both peptides are capable of assembling into amyloid fibrils in vivo, although Aβ_42_ is the more aggregation prone [97]. Fibrils of Aβ_40/42_ deposit in the brains of Alzheimer's sufferers, causing the cell death associated with memory loss [98,99]. It has been suggested that the variation in amyloidogenicity between Aβ_40_ and Aβ_42_ could result from differences in their oligomerization pathways [91,100]. Different oligomers of each peptide, identified using cross-linking and native polyacrylamide gel electrophoresis [100], have been characterized using conventional IMS–MS [91]. Using this approach, Aβ_42_ was found to form both a tetramer and a hexamer. The hexamer was shown to be capable of stacking with a second hexamer to form a 2-ring dodecamer, which then proceeded to form fibrils. In contrast, under the same conditions Aβ_40_ was found to form only a tetramer that was more compact than the Aβ_42_ tetramer. From these data, the authors suggested that the aggregation pathways of Aβ_40_ and Aβ_42_ diverge, resulting in different kinetics and assembly mechanisms for each sequence, commensurate with their different amyloidogenicities.

**Fig. 4:**
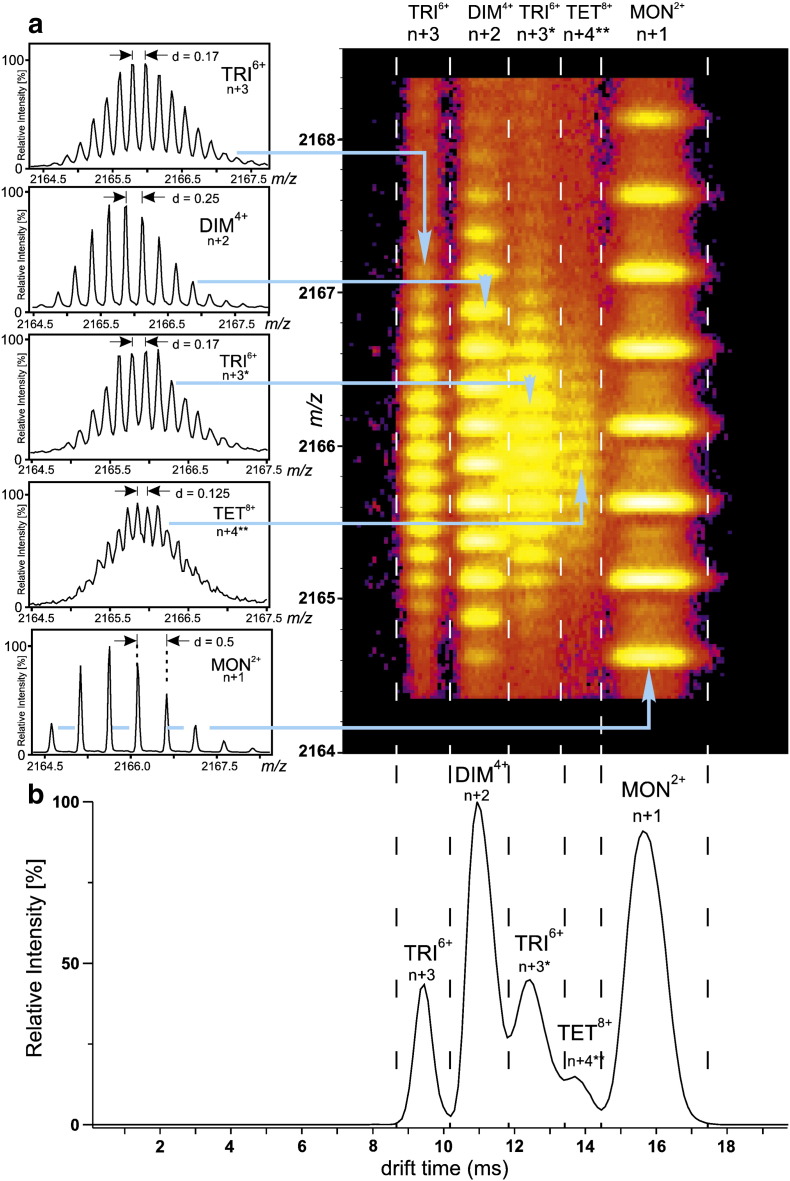
Aβ_40_ oligomers separated and identified by travelling-wave IMS–MS [90]. (a) Three-dimensional IMS–MS spectrum of Aβ_40_ for the *m*/*z* 2164–2168 region containing overlapping charge states arising from monomeric (MON), dimeric (DIM), trimeric (TRI) and tetrameric (TET) Aβ_40_. *m*/*z* values are shown on the vertical axis and ion mobility drift times (ms) on the horizontal axis. The signal amplitude is colour-coded, increasing from purple (low intensity) to bright yellow (high intensity). The insets show the isotopic patterns of the IMS-separated + 2 monomer ions, + 4 dimer ions, + 6 trimer ions and + 8 tetramer ions, all of the same *m*/*z* value, from which the oligomers can be identified unambiguously. Note there are two trimers with different drift-times, the one with the higher mobility and shorter drift time being the more compact of the two. (b) Summary of the oligomer composition of the *m*/*z* 2164–2168 region following assignment of each species based on its C^13^ isotope distribution, demonstrating the presence of two different Aβ_40_ trimers and showing clearly the drift times (ms) of each oligomeric state.

Reprinted with permission from M. Kłoniecki, A. Jabłonowska, J. Poznański, J. Langridge, C. Hughes, I. Campuzano, K. Giles, M. Dadlez, Ion mobility separation coupled with MS detects two structural states of Alzheimer's disease Aβ1–40 peptide oligomers, J. Mol. Biol., 407 (2011) 110–124 [90].

The Aβ_40/42_ results thus described were obtained using negative mode ESI–MS. More recent experiments using positive mode ESI with travelling-wave IMS–MS produced different results [90]. In this case, an array of oligomeric species from monomer to 13-mer inclusive, together with higher order species, were observed for Aβ_40_ [90] (Fig. 4). The discrepancies between the two datasets could arise for a number of different reasons, including the differences in ionization mode (whereby the charges imposed could influence the structure of oligomers), instrument design and variations in sample preparation. All of these variables may lead to differences in the oligomer species detected, especially as it has been reported that several different polymorphs of Aβ_40_ exist [101]. However, analyses of α-synuclein have shown that the ionization mode polarity has no effect on the distribution of monomeric conformers of this protein [54,77,102], nor on the distribution of monomer and dimer species formed [53,103].

The Aβ_40_ study above not only demonstrates the utility of travelling-wave IMS–MS to separate a multitude of Aβ_40_ oligomers, but also highlights the ability of this technique to characterize different conformations of individual oligomeric state [90]. This study used a second-generation travelling-wave IMS–MS instrument with improved *m*/*z* resolution, which allowed charge states to be assigned for highly-charged ions using their C^13^ isotope patterns (Fig. 4).

**Fig. 5:**
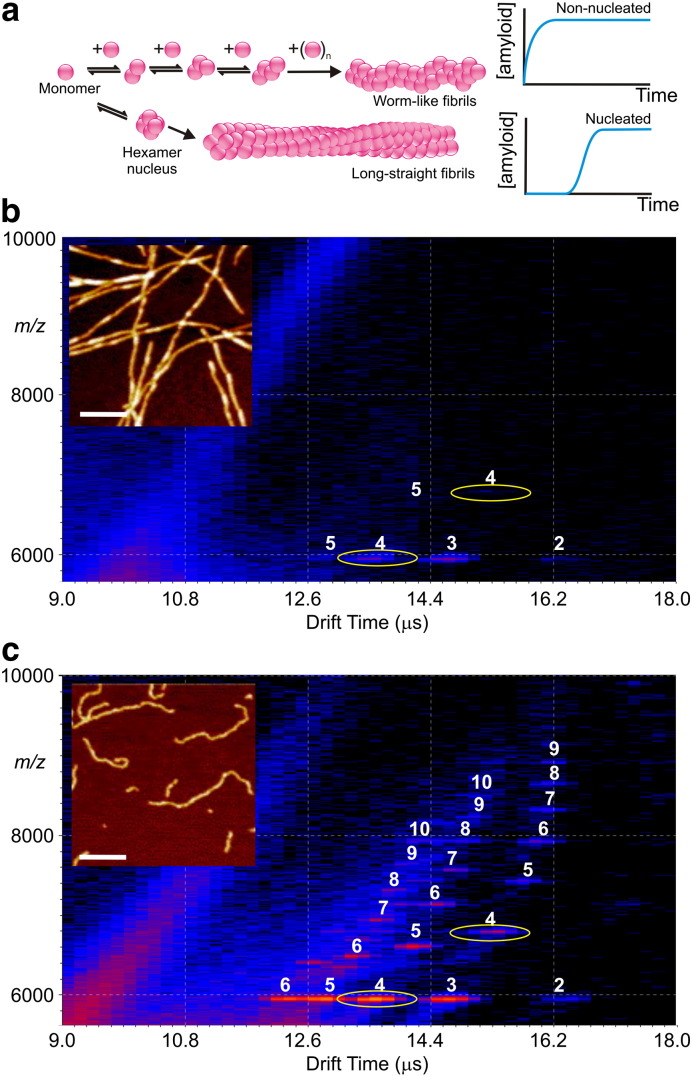
In vitro fibril formation from β_2_-microglobulin (β_2_m) monitored in real-time by travelling-wave IMS–MS. (a) Schematic showing two assembly pathways which diverge to form worm-like fibrils (non-nucleated assembly) or long-straight fibrils (nucleated assembly following a lag-phase). (b) Three-dimensional IMS–MS spectrum (*m*/*z* 6000–10,000) showing β_2_m oligomers 1 min into fibril assembly in 100 mM ammonium formate buffer (pH 2.5), which triggers the formation of long-straight fibrils. (c) Three-dimensional IMS–MS spectrum (*m*/*z* 6000–10,000) showing β_2_m oligomers 1 min into fibril assembly in 400 mM ammonium formate buffer (pH 2.5) which triggers the formation of worm-like fibrils. In both (b) and (c) the numbers above each peak indicate the oligomer size. The + 7 and + 8 charge state ions of the tetramer are highlighted (yellow ovals) in each case to show the increase in their population with increasing ionic strength denoting the point at which the pathways of assembly are thought to diverge. Insets show atomic force microscopy images of the fibrils formed at the end-points of assembly in each case; the scale bars represent 200 nm.

Using ESI and travelling-wave IMS–MS, oligomeric species have been detected during β_2_m fibril formation. β_2_m is able to form fibrils of two distinct morphologies at low pH (Fig. 5a) in vitro [104]. ESI–IMS–MS has been used as a tool to identify the different populations of oligomers formed on each assembly route and to discern where the two different assembly pathways diverge [94]. At pH 2.5 and in low ionic strength buffer, β_2_m forms fibrils in vitro that have a long-straight, unbranched morphology typical of ex vivo amyloid [67,105]. By contrast, at pH 3.6 and with increased ionic strength, β_2_m forms fibrils of a different, less well-ordered morphology, termed “worm-like”, due to their short, curved appearance (Fig. 5a) [89,104,106]. ESI–IMS–MS revealed differences in the distribution of oligomers formed under each of these conditions: whereas oligomers up to tetramer in size, but no larger species, are populated during the formation of long-straight fibrils [93], high-order oligomers up to at least 14-mer are observed during worm-like fibril formation [94] (Fig. 5b, c). These results correlate well with earlier ESI–MS experiments, whereby oligomeric species up to 20-mer in size were identified under conditions that result in worm-like fibrils, while species up to only tetramer were observed under conditions in which long-straight fibrils form [89]. Through comparison of the CCSs of species common to both aggregation mechanisms, the point of divergence of the two pathways has been identified [94]. Although the CCS values for the monomer, dimer and trimer are comparable under both sets of conditions, β_2_m populates an additional, more compact tetramer under conditions that lead to the formation of worm-like fibrils (Fig. 5c). This compact tetramer is thought to oligomerize further by monomer addition, resulting eventually in worm-like fibril formation via an extensive series of oligomers differing in mass by a single monomer (Fig. 5a). By contrast, at pH 2.5 the observation of species only up to a tetramer in size is consistent with a nucleated mechanism for the formation of long-straight fibrils via a hexameric nucleus (Fig. 5a) [43]. Estimation of the CCSs of oligomers formed during the assembly of β_2_m monomers into fibrils has yielded further insights into how these fibrils form. The β_2_m oligomers are consistent with the population of a stacked conformation, much extended when compared with a globular protein of a similar mass [93].

**Fig. 6:**
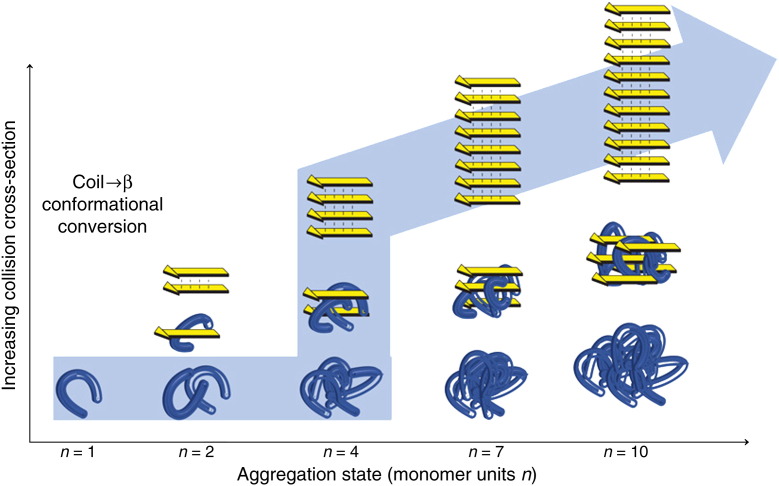
The in vitro self-assembly of peptide monomers into mature, insoluble β-sheet fibrils via an intermediate phase of soluble oligomers monitored by IMS–MS [107]. Self-assembly starts at the folded monomer (n = 1, left) and proceeds to soluble peptide assemblies of increasing mass (n = 2, 4, 7, and 10). Soluble peptide oligomers with identical mass (i.e. the same number of monomer units, n) can assume different conformations such as globular (blue structure, bottom) or β-strand conformations (yellow arrows, top) with different CCS. Successively mass-extracting a specific aggregation state from the solution-phase distribution and subsequent determination of its CCS reveals the self-assembly pathway taking place in solution (large, pale blue arrow). A pronounced transition of soluble oligomers (horizontal axis) from globular conformations into β-strand structures occurs during this phase. The oligomer size at which the divergence from compact to extended conformers occurs differs for peptides of different sequences.

Reprinted with permission from C. Bleiholder, N.F. Dupuis, T. Wyttenbach, M.T. Bowers, Ion mobility–mass spectrometry reveals a conformational conversion from random assembly to β-sheet in amyloid fibril formation, Nat. Chem., 3 (2011) 172–177 [107].

Assembly via extended oligomers has also been shown to occur during the self-assembly of the small peptides NNQQNY, VEALYL and SSTNVG [107], highly-amyloidogenic sequences from the yeast prion protein Sup 35, human insulin, and IAPP, respectively. Aggregation proceeds rapidly in vitro for each of these short sequences, occurring via stacked assemblies of high-order oligomers that populate extended, fibril-like conformers rather than compact or globular assemblies commencing even as early as dimeric species (Fig. 6). These extended oligomers differ dramatically from the oligomers observed for the peptide YGGFL, a non-amyloidogenic control sequence. Interestingly, the amyloidogenic peptides NNQQNY and SSTNVG display initially an isotropic distribution of compact oligomers, but these species divert to extended conformations at the octamer or 11-mer subunit, respectively. By contrast, oligomers of VEALYL populate only an extended conformation initially. These extended oligomers are thought to have β-sheet content postulated to be a prerequisite for amyloid formation. Consistent with these observations, assembly via compact or extended conformers has been predicted using all-atom computer simulations [44], the flux along each pathway depending on the peptide sequence, hydrophobicity and β-sheet propensity.

## 6 ESI–IMS–MS can be used to unravel the mechanism of inhibition of fibril assembly by small molecules

Recently there has been an increasing interest in applying IMS–MS to study small-molecule inhibitors of amyloid systems [95,102,108,109]. While ESI–MS has been used to detect protein-ligand interactions soon after the development of the technique [6,110,111], it was not until over a decade later that this approach was first used to probe inhibitors of amyloid systems [112–114]. One of the earliest studies in this area was performed by Robinson and co-workers who used ESI–MS to identify inhibitors of transthyretin (TTR) amyloidosis [113]. TTR is active in the serum and cerebroserum as a homotetramer, whereby its function is to transport vitamin A or the hormone thyroxine, respectively. However, upon dissociation from its tetrameric state, TTR mis-folds and aggregates into amyloid fibrils. One strategy for preventing TTR amyloidosis is by stabilisation of the native tetramer by ligand binding [113]. These studies showed clear evidence for TTR tetramer stabilisation via binding of ligands that target the thyroxine binding site. Subsequent evolution of these ligands has led to the first small molecule therapeutic for an amyloid disease, tafamidis [115].

**Fig. 7:**
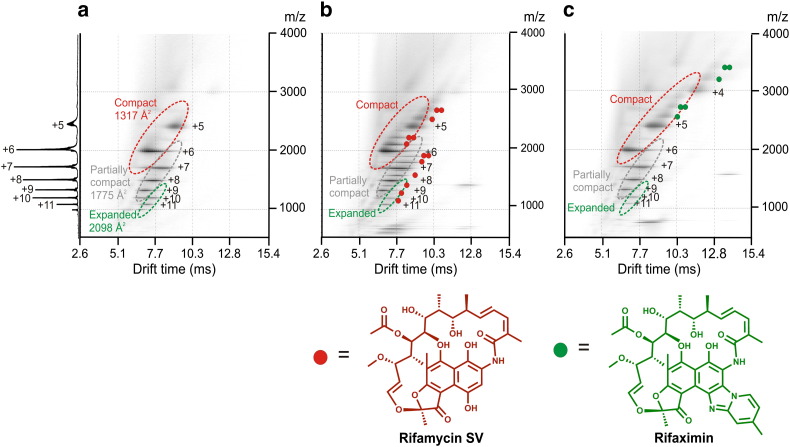
Small molecule inhibition of β_2_m fibril formation in vitro [108]. Three-dimensional IMS–MS spectra of (a) β_2_m monomer alone; (b) β_2_m with equimolar rifamycin SV added; and (c) β_2_m with equimolar rifaximin added. The numbers adjacent to the peaks (e.g. + 5) indicate the charge state carried by those ions, and charge states belonging to the same protein conformers are encircled with dashed lines and labeled individually: compact, partially compact, and expanded. The filled coloured circles on the spectra represent the number of ligand molecules bound to each charge state of β_2_m in the different spectra (red for rifamycin SV, green for rifaximin). (b) Rifamycin SV binds to all charge states and hence all conformers of monomeric β_2_m, thereby inhibiting fibril formation. (c) Rifaximin binds only to the compact protein conformer but not the partially compact or the expanded conformers, and does not inhibit fibril formation. The summed *m*/*z* spectrum for β_2_m (11,860 Da) alone is shown on the left-hand side of (a). The molecular structures of rifamycin SV and rifaximin are positioned below the relevant spectra in (b) and (c), respectively.

Reprinted with permission from L.A. Woods, G.W. Platt, A.L. Hellewell, E.W. Hewitt, S.W. Homans, A.E. Ashcroft, S.E. Radford, Ligand binding to distinct states diverts aggregation of an amyloid-forming protein, Nat. Chem. Biol., 7 (2011) 730–739 [108].

**Fig. 8:**
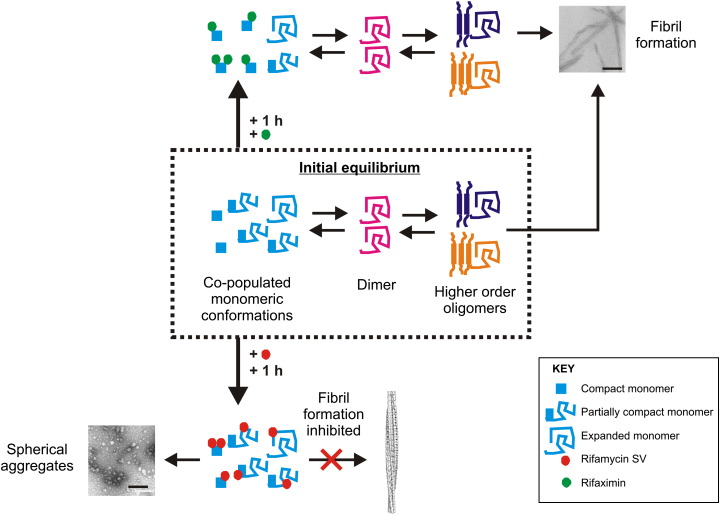
Schematic showing small molecule inhibition of β_2_m fibril formation in vitro [108]. The initial protein equilibrium consists of a mixture of monomeric protein conformers (compact, partially compact, and extended) together with protein dimer and some higher order oligomers. Rifamycin SV binds to the compact, partially compact and expanded conformers of β_2_m, inhibiting fibril formation and diverting assembly to spherical aggregates. Rifaximin, however, binds only to the compact conformer of β_2_m, but not to the partially compact or the extended conformers, and does not prevent β_2_m from undergoing self-aggregation to form fibrils.

Reprinted with permission from L.A. Woods, G.W. Platt, A.L. Hellewell, E.W. Hewitt, S.W. Homans, A.E. Ashcroft, S.E. Radford, Ligand binding to distinct states diverts aggregation of an amyloid-forming protein, Nat. Chem. Biol., 7 (2011) 730–739 [108].

More recent applications of ESI–MS have extended these early analyses, introducing ion mobility separation to determine the mechanism of inhibition when the starting conformer is not a folded state. This approach has now been applied to a variety of such amyloid systems, including Aβ_42_, α-synuclein, and β_2_m at low pH [95,102,108,109]. In these cases, ESI–IMS–MS was used to separate monomers and different oligomers, and to detect inhibitor binding to specific monomeric conformations and oligomeric intermediates. One such study used ESI–IMS–MS to investigate the mechanism of inhibition of β_2_m aggregation into long-straight fibrils at pH 2.5 [108]. In this work, rifamycin SV, an antibiotic from the family of rifamycins (Fig. 7), was shown to be active against β_2_m fibril formation. Interestingly, rifamycin SV also prevents Aβ_40/42_ fibrillation [116]. Rifampicin, a similarly structured macrocyclic antibiotic also of the rifamycin family, has been shown to be active against aggregation of Aβ and α-synuclein [117,118], but was ineffective when incubated with IAPP or β_2_m [108,119]. These results demonstrate that the interaction of rifamycin SV with these proteins is specific, as minor differences in the structure of the small molecule remove its ability to inhibit. Similarly, rifaximin, another antibiotic from the rifamycin family, has been shown to be inactive against β_2_m fibril formation at low pH [108] (Fig. 7). Inactive small molecules that have similar structures to potential inhibitors, and therefore similar electrospray ionization efficiency, are ideal to use as a control for non-specific binding when analysing ligand binding using ESI–MS [120,121]. Binding of rifaximin to β_2_m therefore provides an appropriate control in the determination of the mechanism of action of rifamycin SV. Using ESI-IMS-MS to separate the different monomeric conformers populated by β_2_m at pH 2.5 (Fig. 7a), rifamycin SV was found to bind to all three conformations i.e. compact, partially compact, and extended conformers (Fig. 7b). By contrast, rifaximin binds only to the most compact conformation (Fig. 7c). Previous analyses of β_2_m at acidic pH had indicated that the compact conformation has a CCS that correlates well with that expected for native-like β_2_m [28,93]. Given that fibril formation is not inhibited, but the lag-time of fibril formation is extended in the presence of rifaximin, these data enable the role of different conformers in fibril formation to be deduced (Fig. 8). Accordingly, the most compact conformer is thought not to be necessary for the amyloidogenic pathway. By contrast, the unfolded and partially folded conformers are necessary for assembly into the fibrillar form under these conditions.

In a similar set of experiments, Z-Phe-Ala-diazomethylketone (PADK), a known inhibitor of Aβ_42_ aggregation [122], was analysed by Bowers and coworkers to determine whether any interaction occurs between PADK and Aβ_42_, as well as the effect of the inhibitor on the well-characterized oligomerisation pathway described previously [91,109]. The results obtained were reminiscent of the effect observed between rifamycin SV and β_2_m: IMS–MS revealed that PADK binds to Aβ_42_ monomer and oligomers, the effect of which is to cause the dissociation of high-order oligomers. Thus, PADK binding thwarts Aβ_42_ aggregation by preventing the formation of higher order oligomers and by inducing the dissociation of any previously-formed dodecamers.

The effect of amyloid inhibitors on the conformational properties of monomeric α-synuclein has also been assessed by ESI–IMS–MS in the presence of the inhibitor epigallocatechin gallate (EGCG), a polyphenolic antioxidant abundant in green tea that has also been shown to be effective against Aβ aggregation [102]. The inhibition of fibril formation caused by EGCG has been well characterized, the small molecule being shown to promote the formation of non-toxic, off-pathway aggregates [123,124]. Pukala and co-workers detected a compaction of the amyloidogenic A53T mutant of α-synuclein preceding fibril formation using ESI–IMS–MS [102]. This mutant protein is associated with familial Parkinson's disease and had been analysed previously by others using ESI–IMS–MS [125] and shown to populate a more compact species than wild-type α-synuclein; the conclusion being that this compact amyloidogenic state is associated with increased amyloidogenicity. However, upon addition of the inhibitor EGCG to A53T α-synuclein, the conformational change to the compact, amyloidogenic state was prevented, and instead binding of EGCG to A53T resulted in the formation of a ligand-bound off-pathway conformer [102]. Interestingly, the effect of the small molecule spermine, a compound that accelerates fibril formation of A53T α-synuclein, has also been investigated using ESI–IMS–MS [125]. Spermine was found to bind to α-synuclein, initiating the formation of the compact state of α-synuclein, thereby increasing amyloidogenicity.

Together, these recent experiments demonstrate the significant potential of IMS–MS to identify the mechanism of action of small molecules that modulate the course of fibril assembly. The information gained is powerful, enabling specific protein conformers within an array of unfolded species capable of ligand binding to be identified. In addition, these studies permit the roles of different conformers in the amyloid assembly route to be defined. While structure-based drug design has revolutionized the potentials for therapeutic intervention of native proteins, IMS–MS offers new opportunities for developing and characterizing small molecules able to bind and modulate assembly of highly dynamic, non-native states.

## 7 Outlook and future potentials of IMS–MS in amyloid research

Investigating the mechanism of amyloid fibril formation using mass spectrometry has progressed significantly in the past decade, yet many questions remain to be answered. These enigmas include defining how fibril formation proceeds and the precise protein/peptide interfaces that are involved in oligomerization. Further insights into the structure of oligomers formed during amyloid assembly, and also by dissociation from fibril ends, are key to evaluating and preventing the toxic effects of amyloid formation and deposition. Currently, a number of chemical techniques are being exploited in conjunction with MS and IMS–MS with the aim of providing complementary information to that available using IMS–MS alone. These include hydrogen deuterium exchange–mass spectrometry (HDX–MS), cross-linking, oxidative labeling and chemical modification [11,126–130]. For example, pulsed-labeling HDX–MS has revealed how a structural rearrangement of oligomers precedes fibril formation of an SH3 domain [45], has produced insights into the β-hairpin arrangement of Aβ_40_ and Aβ_42_ oligomers [131] and has shown how Aβ_40/42_ fibrils exist in equilibrium with Aβ monomer in solution [132]. The use of IMS–MS integrated with these analyses would allow modelling of the possible conformations of each of these oligomers. Covalent modification of oligomers can also be used in conjunction with IMS–MS to give more depth to these analyses. Oligomers can be cross-linked by introducing photo-reactive substituents into the protein/peptide sequence or by using other photo-oxidation strategies [133–135]. Activation of these substituents initiates covalent bond formation, thus trapping the intermediate species. These cross-linked species can then be analysed using IMS–MS to yield information about their shape, the site of the cross-link, and therefore the oligomerization interface. Alternatively, oxidative foot-printing can be used to label the amino acid side chains of proteins [126,127], revealing sites that are protected during oligomerization. Further, developments in IMS–MS instrumentation has enabled site-specific information about protein conformation and binding interfaces to be revealed using gas-phase HDX labeling [136,137]. For example, HDX of labile amino acid side-chain protons takes place within the mass spectrometer following the ionization process, through collisions with deuterated ammonia. Electron transfer dissociation can then be used for top-down sequencing [138,139] of the intact protein to reveal site-specific information about the location of exposed side-chains.

In summary, the last decade has witnessed enhancements in ESI–MS by means of integration with IMS for the analysis of the structure, assembly and dynamics of complex macromolecular systems including amyloid fibril formation. The separating power of ESI–IMS–MS, together with the potential of chemical labeling, HDX and other chemical strategies will ensure that ESI–IMS–MS will continue to play a major role in our quest to determine the molecular mechanisms of assembly of peptides and proteins into amyloid fibrils. Perhaps most excitingly, using ESI–IMS–MS to facilitate the design, development and analysis of protein–ligand interactions in amyloid formation offers new opportunities to devise novel therapeutic strategies to inhibit fibril formation and/or the population of cytotoxic species.

## Figures and Tables

Figure adapted from [15].

Figure taken from T. J. Jahn, PhD Thesis, University of Leeds, 2006 [64].

The original data were published in D.P. Smith, L.A. Woods, S.E. Radford, A.E. Ashcroft, Structure and dynamics of oligomeric intermediates in beta2-microglobulin self-assembly, Biophys. J., (2011) 101, 1238–1247 [94].
